# Physical, behavioural, and psychosocial factors associated with childhood and adolescent obesity: a study from the United Arab Emirates

**DOI:** 10.3389/fped.2025.1701873

**Published:** 2025-11-14

**Authors:** Tanveer Ashraf, Alaina L. Pearce, Radwa Helal, Sarah Saidi, Chandan J. Vaidya, Nader Lessan

**Affiliations:** 1 Research Institute, Imperial College London Diabetes Centre, Abu Dhabi, United Arab Emirates; 2 Department of Psychology, Georgetown University, Washington, DC, United States; 3 Department of Nutritional Sciences, The Pennsylvania State University, University Park, PA, United States

**Keywords:** paediatric obesity, sleep, psychosocial health, socio-economic status, obesity

## Abstract

**Aims:**

Paediatric obesity is associated with both physical and psychosocial health. Whether those associations, which are most widely reported in Western samples, are also observed in other sociocultural settings is not well known. This study aimed to characterise lifestyle, physical, and psychological characteristics associated with excess weight in Emirati youth, a population that has not been widely studied.

**Materials and methods:**

Height and weight were measured for 107 youth (46 male; 7–17 years) from Abu Dhabi. A detailed medical history was obtained from the Imperial College London Diabetes Centre. Parents reported relevant family history and demographics, sleep using the Children's Sleep Habits Questionnaire, and mental health problems using the Strengths and Difficulties Questionnaire.

**Results:**

Older youth and those with a family history were more likely to have obesity; however, weight status did not differ by sex, income, or parental education. Anaemia was more prevalent in youth with a healthy weight, while hyperlipidemia was more prevalent in youth with overweight or obesity. Obesity was associated with disordered breathing during sleep but not with sleep times or duration. Overall, weight status did not relate to mental health problems; however, obesity was associated with a greater risk for peer problems in females but not males.

**Conclusions:**

These results highlight factors that may play a role in culturally specific drivers of paediatric obesity in the Middle East and will help to natively tailor prevention and intervention efforts.

## 1 Introduction

The growing burden of obesity in children and adolescents is a significant public health concern worldwide (1). The prevalence of overweight and obesity among 5–19-year-olds has risen from 4% in 1975 to over 18% in 2016 (2). Similarly, rates of paediatric obesity are increasing in Gulf Cooperation Council (GCC) countries, including the United Arab Emirates (UAE), Saudi Arabia, Bahrain, Qatar, Oman, and Kuwait (3), with the UAE Ministry of Health and Prevention reporting 17.4% of 5–17-year-old children and adolescents had obesity in 2019 (4). However, the lack of uniformity in reference standards and reporting systems renders comparisons difficult (3). Children with obesity face a lifelong burden of deleterious comorbidities, with obesity contributing to over one-third of adult non-communicable disease incidence, including cardiovascular diseases, cancers, chronic respiratory disorders, type 2 diabetes mellitus, and mental health disorders (5). The economic burden of obesity, with the annual global cost of childhood obesity projected to increase from $13.62 billion to $49 billion by 2050 (6). Therefore, early prevention and intervention are necessary, which requires a better understanding of the factors that contribute to the development and maintenance of excess weight in childhood.

The aetiology of childhood obesity is multifactorial, including behavioural, sociocultural, environmental, and genetic contributions, which complicate strategies for its prevention and management (7–9). Additionally, the current understanding of the factors associated with paediatric obesity largely stems from Western studies, where socioeconomic disparities remain a key determinant of obesity risk across countries. For example, lower socioeconomic status and paediatric obesity are both independently associated with cognitive, sleep, and mental health problems (9–12). Examination of these associations in countries with different socioeconomic and sociocultural structures is needed to understand the contributing factors and inform the management of excess adiposity and its consequences (13). Recent reviews and meta-analyses of risk factors for childhood obesity in GCC found that the risk for obesity was greater in older children, in children whose parents and/or siblings had elevated body mass index (BMI), in children with lower physical activity, and in children who ate out at restaurants more than twice a week and consumed more fast food (3, 14). However, the review noted inconsistencies across studies and countries. To better understand the factors that contribute to paediatric obesity in the UAE, more comprehensive studies are needed that assess familial, lifestyle, and medical histories.

There have been calls for more research on the biological, environmental, and cultural drivers of obesity in the Gulf region (15). Therefore, this study examined the associations of weight status with demographic characteristics, sleep and physical health, and mental health in Emirati children and adolescents living in Abu Dhabi.

## 2 Materials and methods

### 2.1 Participants

A sample of 107 children and adolescents (61 female, 46 male) aged 7–17 years old were recruited from the Imperial College London Diabetes Centre (ICLDC) in Abu Dhabi, UAE, from 2019 to 2020. Clinic patients were invited to participate if they were not currently diagnosed with type 2 diabetes. Using the International Obesity Task Force (IOTF) criteria (16), 41 (38%) had a healthy weight, 29 (27%) had overweight, and 37 (35%) had obesity (see Table 1 for participant characteristics). Participants were excluded if they were diagnosed with a neuropsychiatric disorder, were currently taking any psychotropic medications, had a learning disability or difficulty that would make completing the study protocol difficult, or had a history of or current seizure disorder or use of neuroleptic medications. According to the Institutional Review Board at ICLDC, informed consent and assent were obtained before participation.

**Table 1:** Participant characteristics by weight status.

	Healthy weight (*n* = 41)	Overweight (*n* *=* 29)	Obese (*n* *=* 37)
Sex, *n* (%)			
Male	17 (59%)	14 (48%)	23 (62%)
Female	24 (41%)	15 (52%)	14 (38%)
Age, years	11.85 [8.02–17.37]	12.84 [8.15–17.54]	13.69 [7.31–17.84]
Body Mass Index	17.15 [12.71–22.72]	24.03 [18.70–28.86]	35.08 [21.87–55.52]
Percent of Overweight	80.86% [64–98]	109.66% [100–121]	155.80% [122–239]
Father Education, yr	12.79 [6–18]	13.60 [6–18]	12.20 [0–18]
No Response	3	0	2
Mother Education, yr	13.38 [3–18]	13.93 [9–18]	12.40 [0–18]
No Response	4	2	2
Monthly Income			
>75,000 AED	4 (11%)	3 (10%)	2 (6%)
55,000–75,000 AED	2 (5%)	3 (10%)	1 (3%)
25,000–55,000 AED	21 (57%)	13 (45%)	19 (58%)
<25,000 AED	10 (27%)	10 (34%)	11 (33%)
No Response	4	0	4
Father Nationality			
Emirati	40 (100%)	25 (96%)	33 (94%)
Omani	0 (0%)	1 (4%)	0 (0%)
Yemeni	0 (0%)	0 (0%)	2 (6%)
No Response	1	3	2
Mother Nationality			
Emirati	38 (93%)	26 (93%)	32 (86%)
Omani	0 (0%)	1 (4%)	0 (0%)
Yemeni	0 (0%)	0 (0%)	1 (3%)
Moroccan	1 (2%)	0 (0%)	1 (3%)
Egyptian	2 (5%)	0 (0%)	1 (3%)
Bahrani	0 (0%)	1 (4%)	0 (0%)
No Response	0	1	2

Percent of Overweight was calculated: 100 × BMI/IOTF-25; IOTF-25: International Obesity Task Force age- and sex-adjusted BMI 25 at age 18.

Mean and range presented for all continuous data and *N* (%) presented for categorical data.

Percent values may not add 100% due to rounding.

#### 2.1.1 Power analysis

Based on findings from a US sample of a similar age range (7–18 years old), the anticipated effect size for the association between weight status and sleep health and mental health is medium-large (*r* = 0.48) (17) and small-medium (*r* = 0.22), respectively. The present study was powered to detect associations between %IOTF-25 and sleep health (0.90 power) and to detect main effects and interactions between %IOTF-25 and sex for mental health (0.73 power).

### 2.2 Protocol

Participants completed the study on the same day of their visit to ICLDC. Parents completed questionnaires pertaining to their family demographics, history of obesity and eating disorders, and their child's sleep and mental health. Youth height and weight were measured, and children and adolescents also completed a series of cognitive assessments (reported in Pearce et al.). Participants' medical histories at the ICLDC were reviewed to identify medical conditions (medical record data collected at the time of participant from 2019 to 2020). A trained researcher administered all surveys in Arabic to the parents.

#### 2.2.1 Anthropometrics

**Figure 1:**
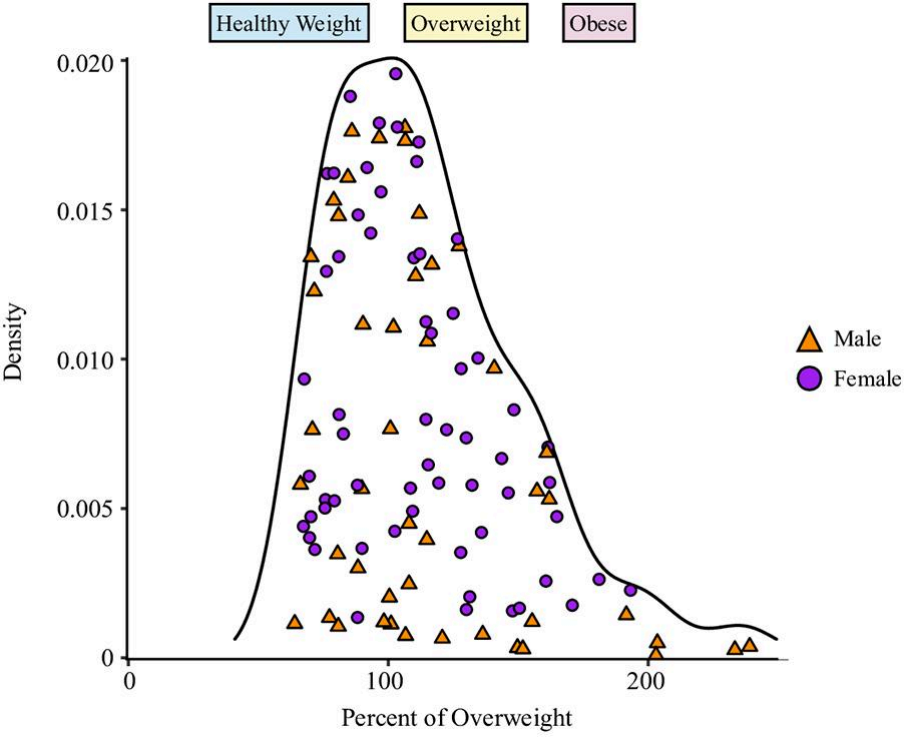
Density distribution of percent of overweight. The shaded regions indicate weight status: healthy weight = blue, overweight yellow, obese = red. Sex is indicated by shape and colour: male = orange triangle, female = purple circle.

Height and weight were recorded twice on the same day, once by a qualified nurse and once by trained researchers at the researcher using a standard scale (Tanita Japan) and stadiometer (Seca). Weight status was classified using the IOTF age- and sex-adjusted cut-offs (16). Percent of overweight was calculated by dividing BMI by the age- and sex-adjusted overweight cut-off $\left(100\times \frac{\mathrm{BMI}}{\mathrm{IOTF}\;\mathrm{BMI}-25};\text{\%}\mathrm{IOTF}-25\right)$. Therefore, values less than 100% indicate a healthy weight, a value of 100% indicates the participant is overweight and his/her BMI is right at the overweight cut-off, and values over 100% indicate overweight or obesity (see Figure 1). Percent of overweight has shown to have a more appropriate distribution (18) and better correspondence with measured body circumferences and fat mass than BMI-z/BMI-SDS or BMI-percentile in samples with moderate (19) and severe obesity (19, 20).

#### 2.2.2 Demographics and family history

Parents completed a demographic questionnaire asking them to report their ethnicity, years of education, and monthly household income. In addition to demographic information, parents also reported whether any members of the family had a history of or currently have obesity or eating disorders (e.g., anorexia, binge eating) and to mark which categories of family relationships (e.g., mother, brother, uncle) had a history or currently have the respective condition. This provides an index of the extent to which a family has been impacted by obesity and/or eating disorders.

#### 2.2.3 Sleep behaviours

The Children's Sleep Habits Questionnaire (CSHQ) is a validated screening instrument used to identify behavioural sleep problems in children (21). As part of the CSHQ, parents also reported their child's typical bedtime and waketime, which was used to compute duration in bed for each participant. Additionally, participants were categorised according to whether their reported bedtime and total sleep duration met current international sleep recommendations for school-aged children and adolescents (22, 23).

#### 2.2.4 Mental health

The Strengths and Difficulties Questionnaire (SDQ) is a widely validated instrument for assessing emotional and behavioural difficulties in children across diverse cultures (24, 25). Parents completed the Arabic version of the SDQ, validated for Arabic-speaking populations, which measures emotional, conduct, hyperactivity, and peer problems, as well as prosocial behaviours (26). To examine risk for mental health-related issues, the four-band categorisation was reduced to two categories: typical (i.e., “Close to Average”) and at-risk behaviour (i.e., “Slightly Raised/Lowered” to “Very High/Low”) (27).

### 2.3 Analyses

#### 2.3.1 Analytic approach

All analyses were conducted in R (28) with data and code available at https://osf.io/4w725/.

For demographic characteristics and sleep behaviour, associations with %IOTF-25 were tested using Pearson's correlation for continuous outcomes (e.g., years of maternal education, age). To test mean differences in %IOTF-25 between groups, *t*-tests or one-way analyses of variance (ANOVA) were used (e.g., sex, income category). We used Fisher's exact *p*-value to test the prevalence of different medical comorbidities across weight status categories. For medical comorbidities observed in more than 20 participants, *t*-tests were used to test for mean differences in %IOTF-25 between those with and without the comorbidity. For count data (e.g., number of family members), associations with %IOTF-25 were assessed using Poisson regressions. Given well-established global differences in the prevalence of mental disorders between males and females (29), logistic regressions were used to examine potential sex × %IOTF-25 interactions in the odds of experiencing behavioural problems or low prosociality (see 2.2.3 Mental Health). Due to the inclusion of an interaction, %IOTF-25 was centered at 100% for these models.

Sensitivity analyses were conducted for all significant associations with %IOTF-25 to test the stability of the effect after accounting for individual differences in sex, age, and socio-economic status, which was indexed by monthly income and maternal education. Additional sensitivity analyses were conducted for family history, sleep, and mental health models after removing youth with Growth/Stature co-morbidities (*n* = 10; Table 2). Removal of these youth did not change the patterns of results for any models.

**Table 2:** Medical comorbidities by weight status.

	Healthy weight	Overweight	Obese
Vitamin D Deficiency	36 (88%)	29 (100%)	33 (89%)
Anaemia			
Iron Deficiency Anaemia (ID)	9 (22%)	7 (24%)	5 (14%)
Thalassemia Minor (TM)	2 (5%)	1 (3%)	0 (0%)
G6PD Deficiency	0 (0%)	0 (0%)	0 (0%)
ID and TM	1 (2%)	1 (3%)	0 (0%)
ID and G6PD Deficiency	1 (2%)	0 (0%)	0 (0%)
Unspecified	9 (22%)	1 (3%)	2 (5%)
Hyperlipidaemia	0 (0%)	5 (17%)	7 (19%)
Thyroid Conditions			
Abnormal Function	5 (12%)	4 (14%)	5 (14%)
Autoimmune Thyroiditis	1 (2%)	2 (7%)	2 (5%)
Unspecified Hypothyrodism	1 (2%)	2 (7%)	2 (5%)
Goiter	0 (0%)	1 (3%)	0 (0%)
Glycaemic Status			
Impaired Fasting Glucose	4 (10%)	8 (28%)	7 (19%)
Impaired Glucose Tolerance Test	2 (5%)	2 (7%)	3 (8%)
Type—I Diabetes	1 (2%)	1 (3%)	0 (0%)
Acanthosis nigricans	1 (2%)	0 (0%)	7 (19%)
Hypertension			
Essential Primary Hypertension	0 (0%)	0 (0%)	2 (5%)
High Blood Pressure	0 (0%)	0 (0%)	1 (2%)
Growth/Stature			
Failure to Thrive (FT)	1 (2%)	0 (0%)	0 (0%)
Growth Hormone Deficiency	1 (2%)	0 (0%)	0 (0%)
Short Stature	3 (7%)	1 (3%)	2 (5%)
FT and Short Stature	1 (2%)	0 (0%)	0 (0%)
Short Stature and Precoscious Puberty	1 (2%)	0 (0%)	0 (0%)
Polycystic Ovarian Syndrome			
Polycystic Ovarian Syndrome	1 (2%)	0 (0%)	1 (2%)
Hirsutism	0 (0%)	0 (0%)	1 (2%)
Hirsutism and Unspecified Ovarian Cysts	1 (2%)	0 (0%)	0 (0%)

Percent values reflect the percent of participants for each weight status categories. Percent values do not add to 100% because participants can have more than 1 condition and only the number with each condition are listed.

## 3 Results

### 3.1 Participant characteristics

This sample ranged in BMI from 11.85 to 55.52 kg/m^2^ with all weight categories fairly evenly represented (using IOTF criteria) (16): healthy weight: 38%, *n* = 41; overweight: 27%, *n* = 29; obese: 35%, *n* = 37. Of those with obesity, 62% (*n* = 23) had morbid obesity, which was defined as a BMI greater or equal to the IOTF age- and sex-adjusted BMI of 35 at 18 years (BMI-35) (16). %IOTF-25 had a well-balanced, continuous distribution (Figure 1), which ranged from 63.95% to 239.00% (Figure 1 and Table 1).

IOTF-25 did not differ by sex [*t*(79) = −072, *p* *=* 0.471] or monthly income [F(3, 95) = 0.24, *p* *=* 0.867] and was not associated with maternal (*r* = −0.15, *p* *=* 0.131) or paternal (*r* = −0.07, *p* *=* 0.491) education. There was, however, a significant association between %IOTF-25 and age (*r* = 0.26, *p* *=* 0.006), indicating older participants tended to have higher %IOTF-25. The follow-up sensitivity test (see 2.3.1) showed that after adjusting for covariates, age was positively associated with %IOTF-25 such that for each year older, a youth's percent of overweight is expected to be 4.24 percentage points higher [*β*(se) = 4.24 (1.46), *p* *=* 0.005]. This indicates the degree of excess weight was greater in older than younger participants in this sample.

### 3.2 Medical comorbidities

Overall, the number of medical comorbidities ranged from zero to four with an average of two comorbidities per participant (mean = 2.15, SD = 1.04), not including overweight/obesity. The number of comorbidities was not associated with %IOTF-25 [*β*(se) = 0.001 (0.002), *p* *=* 0.598] and did not differ by sex [*t*(101) = 1.13, *p* *=* 0.263] (Table 2).

### 3.3 Family history of obesity and eating disorders

Overall, 70% (*n* = 70 of 101) of parents reported at least one family member with a history of or present obesity. %IOTF-25 was greater if the family reported a history of obesity (mean = 125.09%, SD = 38.38%) than if they did not [mean = 94.77%, SD = 23.87%; *t*(87.9) = 4.83, *p* < 0.001]. The number of different family relationship categories selected as having a history of obesity was positively associated with %IOTF-25 [*β*(se) = 0.009 (0.002), *p* < 0.001] such that a youth with 110% of overweight would have 9% greater odds of having an additional family relationship category with a history of obesity compared to a youth at 100% of overweight $\left(e^{\beta *10}=\;e^{0.09}=1.09\right)$. A sensitivity test showed %IOTF-25 remained significant after adjusting for covariates [*β*(se) = 0.008 (0.002), *p* < 0.001].

In contrast, fewer families reported a history of eating disorders (11%, *n* = 11 of 96). Due to the limited number of participants with a family history, differences in %IOTF-25 could not be tested. However, the prevalence of a family history did not differ across weight statuses (Fisher's *p* *=* 0.263). When asked to indicate which family relationship categories had or currently have an eating disorder, the number of categories selected ranged from one to seven across the 11 participants who reported a family history (overall sample mean = 0.21, SD = 1.75). For those that reported a history (*n* = 11), the child's aunt was selected 64% of the time (*n* = 7), grandmother was selected 36% of the time (*n* = 4), uncle was selected 27% of the time (*n* = 3), brother, father, sister, and grandfather were each selected 18% of the time (*n* = 2) and mother was selected 9% of the time (*n* = 1).

### 3.4 Sleep behaviour

The average bedtime reported was 22:14 (SD = 1.86 h). Reported waketimes ranged from 05:00 (5 am) to 15:30 (3:30 pm) with an average waketime of 06:52 (SD = 1.79 h). Time spent in bed ranged from 2 to 12 h with an average of 8.46 h (SD = 1.65 h). Overall, 53% (*n* = 52 of 47) of participants met the recommendation to have a bedtime within the 21:00/9 pm hour (22), and 44% (*n* = 43 of 97) met the recommendations for total sleep time (9–11 h for 7–12-year-olds and 8–10 h for 13–17-year-olds) (23). %IOTF-25 was not associated with bedtime (*r* = 0.13, *p* *=* 0.192), time in bed (*r* = −0.09, *p* *=* 0.392), or waketime (*r* = 0.06, *p* *=* 0.584) and did not differ if sleep duration [*t*(96) = 0.50, *p* *=* 0.615] or bedtime [*t*(85) = 1.68, *p* *=* 0.098] recommendations were met (Table 3).

**Table 3:** Sleep and mental health behaviours.

	Healthy weight	Overweight	Obese
Children's sleep habits questionnaire			
Bedtime, M [range] adjusted to a 28 h day^a^	21:62 [19:00–25:00]	22:66 [20:00–27:30]	22:60 [19:00–28:00]
Waketime, 24 h time, M [range]	06:53 [05:00–10:50]	07:08 [05:30–15:50]	07:09 [05:00–14:00]
Time in Bed, hours, M [range]	8.68 [2.00–11.50]	8.48 [5.00–12.00]	8.21 [6.00–11.00]
Bedtime Resistance (max = 15), M (SD)	7.77 (2.41)	6.91 (1.83)	7.19 (2.08)
Sleep Onset Delay (max = 3), M (SD)	1.51 (0.68)	1.62 (0.85)	2.09 (0.92)
Sleep Duration (max = 9), M (SD)	4.55 (1.62)	4.52 (1.85)	4.89 (1.70)
Sleep Anxiety (max = 12), M (SD)	6.13 (2.51)	5.76 (2.52)	5.14 (2.10)
Night Waking (max = 6), M (SD)	2.89 (1.41)	2.75 (0.99)	3.09 (1.29)
Parasomnias (max = 21), M (SD)	8.67 (2.33)	8.58 (1.50)	9.21 (2.61)
Disordered Breathing (max = 9), M (SD)	3.35 (1.11)	3.65 (1.34)	4.52 (1.88)
Daytime Sleepiness (max = 22), M (SD)	13.14 (4.09)	11.67 (3.31)	12.52 (2.80)
Strengths and Difficulties Questionnaire			
Emotional Problems, *n* (%)			
Close to Average	24 (62%)	18 (64%)	20 (54%)
Slightly Raised	5 (13%)	3 (11%)	6 (16%)
High	7 (18%)	5 (18%)	7 (19%)
Very High	3 (8%)	2 (7%)	4 (11%)
Conduct problems, *n* (%)			
Close to Average	26 (65%)	21 (75%)	22 (65%)
Slightly Raised	6 (15%)	3 (11%)	4 (12%)
High	7 (18%)	4 (14%)	7 (21%)
Very High	1 (3%)	0 (0%)	1 (3%)
Hyperactivity Problems, *n* (%)			
Close to Average	29 (74%)	24 (89%)	32 (86%)
Slightly Raised	8 (21%)	1 (4%)	3 (8%)
High	1 (3%)	0 (0%)	0 (0%)
Very High	1 (3%)	2 (7%)	2 (5%)
Peer Problems, *n* (%)			
Close to Average	17 (45%)	12 (41%)	11 (31%)
Slightly Raised	7 (26%)	6 (21%)	7 (20%)
High	10 (26%)	9 (31%)	11 (31%)
Very High	4 (11%)	2 (7%)	6 (17%)
Prosocial, *n* (%)			
Close to Average	30 (77%)	22 (79%)	25 (71%)
Slightly Lowered	5 (13%)	4 (14%)	6 (17%)
Low	2 (5%)	2 (7%)	1 (3%)
Very Low	2 (5%)	0 (0%)	2 (9%)

a Since some bedtimes occurred between 00:00–04:00 (12–4 am), the daylength was adjusted to 28 h so that midnight = 24:00 and 04:00/4 am = 28:00.

Percent values may not add 100% due to rounding.

M, mean; SD, standard deviation.

Although %IOTF-25 was not associated with sleep duration or typical sleep patterns, it was associated with CSHQ subscales Sleep Onset Delay (*r* = 0.31, *p* *=* 0.002) and Sleep Disordered Breathing (*r* = 0.37, *p* < 0.001). No other CSHQ subscales were associated with %IOTF-25 (*r*'s = −0.18–0.10, *p*'s = 0.070–0.692). Sensitivity test adjusting for covariates (see 2.3.1) showed %IOTF-25 was positively associated with both Sleep Onset Delay [*β*(se) = 0.006 (0.003), *p* *=* 0.037] and Sleep Disordered Breathing [*β*(se) = 0.016 (0.005), *p* *=* 0.002]. After accounting for covariates, a youth with 110% of overweight, relative to 100% overweight, would be expected to have a 0.06-point higher Sleep Onset Delay score (range 0–3) and 0.12-point higher Sleep Disordered Breathing score (range 3–9). Together, this suggests that while overall sleep patterns do not differ by percent of overweight, youth with higher weight status are more likely to take longer to fall asleep and have more sleep-related disordered breathing (e.g., snoring, snorts/gasps) (21).

### 3.5 Mental health

**Figure 2:**
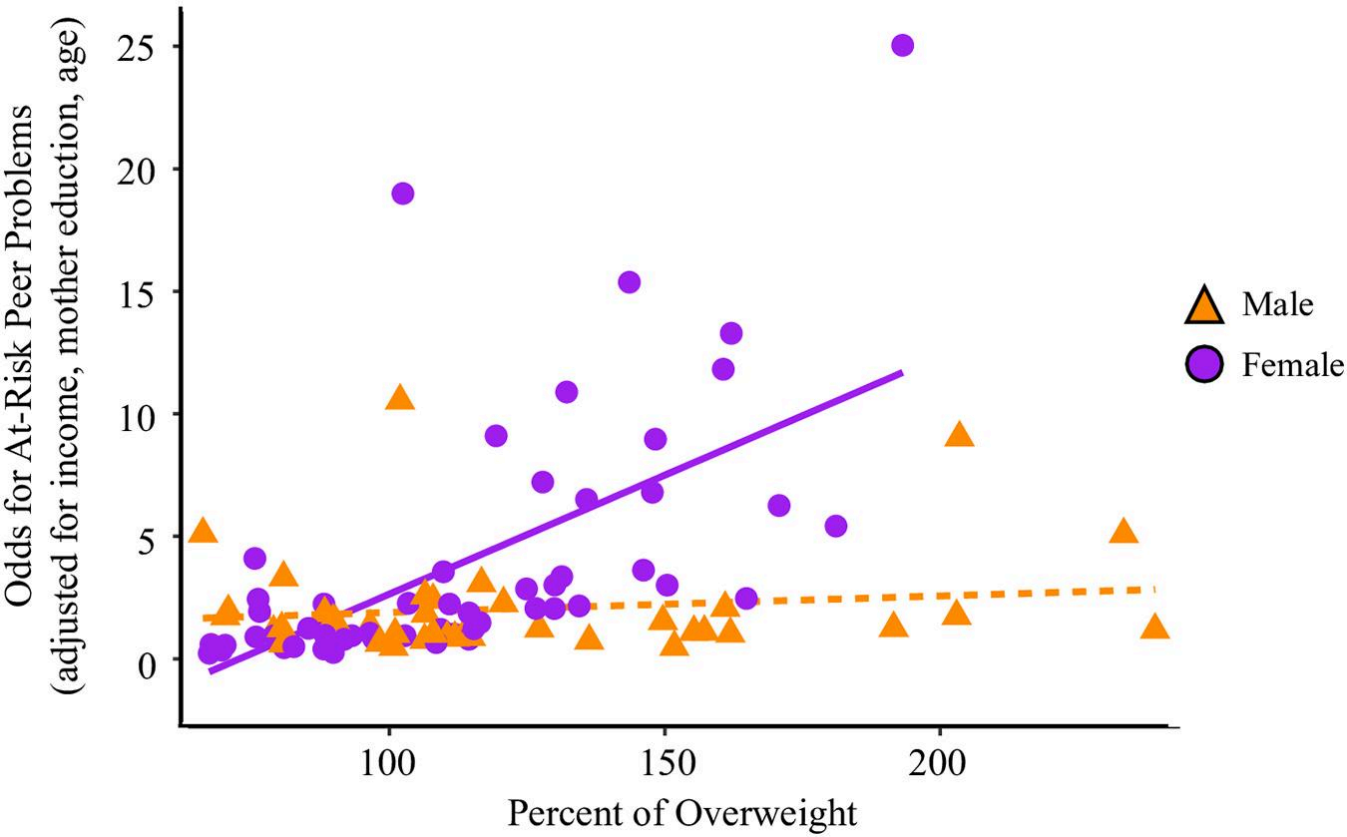
Peer problems sex × percent of overweight. This figure shows the predicted odds of experiencing At-Risk peer problems by sex and percent of overweight after adjusting for monthly income, maternal education, and age. The shaded regions indicate the standard error region around each slope. Sex is indicated by shape and colour: male = orange triangle, female = purple circle.

There was no evidence for elevated mental health problems in this sample, with the majority of participants experiencing typical or “Close to Average” behavioural profiles for the primary mental health dimensions (emotional: 60%, *n* = 62; conduct: 68%, *n* = 69; hyperactivity: 83%, *n* = 85) and prosociality (75%, *n* = 77). After adjusting for all covariates, logistic regressions showed no significant associations with %IOTF-25 or sex-by-%IOTF-25 interactions for the likelihood of experiencing at-risk levels for emotional, conduct, hyperactivity, or prosocial problems (*p*'s > 0.055). Relative to the other SDQ subscales, the proportion of participants with typical or “Close to Average” peer problems was lower (39%, *n* = 40). Sensitivity tests (see 2.3.1) showed a significant sex × %IOTF-25 interaction for peer problems [*β*(se) = −0.030 (0.014), *p* *=* 0.036; Figure 2]. *post-hoc* tests showed males had no association between %IOTF-25 and odds of being at-risk for peer problems [*β*(se) = −0.006 (0.009), 95% CI: −0.02–0.01] while females had a significant positive association [*β*(se) = 0.024 (0.012), 95% CI: 0.001–0.046]. Thus, while males did not show increased risk for peer problems with greater weight status, a female youth with 110% overweight had 27% greater odds of having peer problems than a female youth with 100% of overweight $\left(e^{\beta *10}=\;e^{0.24}=1.27\right)$ (Table 3).

## 4 Discussion

This is a preliminary, comprehensive examination of the association between physical, behavioural, and psychosocial characteristics and the weight status of Emirati youth in the UAE. Prevalence of obesity was higher in older youth and in those having more family members with obesity; however, obesity was not associated with sex, parental education or family income. Among the many physical health conditions observed, only two were associated with weight status: anaemia was more prevalent in youth with a healthy weight, while hyperlipidemia was more prevalent in youth with overweight or obesity. Among sleep characteristics, only delayed sleep onset and disordered breathing were associated with obesity. Lastly, there was no evidence of elevated behavioural problems regarding attention/hyperactivity, conduct, emotional, and social behaviour in youth with obesity. However, girls with obesity experienced more problems with peer relations than their male counterparts. Overall, this study identified preliminary health and behavioural risk factors in Emirati youth with obesity. These findings provide a foundation to generate hypotheses that can be tested in larger samples of Emirati youth. Overall, these results highlight the need for directing culturally tailored prevention and intervention efforts.

To better understand the physical complications associated with obesity in Emirati youth, medical records were reviewed. Not considering the medical conditions of overweight or obesity, participants had an average of two medical conditions. Vitamin D deficiency was most prevalent and did not differ by weight status. A high prevalence of Vitamin D deficiency has been noted in the UAE (30, 31) and other Gulf region countries (32) and clinical practice guidelines have been proposed to address this for the UAE (33). However, prevalence of anaemia was greater in youth with healthy weight while prevalence of hyperlipidaemia was greater in those with overweight and obesity. This replicates previous findings from the UAE showing dyslipidaemia in youth with excess weight (34, 35). Together, the reported patterns of medical complications align with previously observed patterns in the UAE indicating good generalizability with other studies of youth in the UAE.

The current study showed both similarities and differences from studies in Western samples. In the current study, there was no association between weight status and family income or parental education level. This is in contrast to patterns seen in Western populations, where youth with lower socioeconomic status experience increased risk for obesity, which suggests that risk for obesity may not be reliably associated with socioeconomic status across different sociocultural settings. Similar to findings in Western samples (36), the prevalence of obesity was associated with current and/or past family history of obesity. Seventy percent of participants had at least one member in their family who currently has obesity or has had obesity in the past and youth with a greater number of relatives with a history of obesity also had higher weight status. Consistent associations between youth obesity and a family history of obesity across studies suggest efforts should be made to identify both heritable and cultural risk factors for obesity.

Although there have been suggestions that obesity is related to sleep disturbances in youth, the lack of well-designed prospective investigations has made it difficult to arrive at firm conclusions for the UAE. While about half the youth met the recommended bedtime, only 44% met the recommendations for total sleep. Sleep recommendations norms are based on studies of Western samples and thus, the observed low rates suggest notable cultural differences in sleep habits. Overall, there was no association between weight status and bedtime, time in bed, or wake time. However, higher weight status was associated with delayed onset of sleep and more sleep-disordered breathing incidents, such as snoring and snorts or gasps, commonly associated with obstructive sleep apnea. This replicates findings from the UAE (37) and Western (17) studies showing worse sleep health and sleep disordered breathing in children and adolescents with obesity. Pediatric obesity has been associated with increased risk for obstructive sleep apnea in both Western (38) and Eastern (39) samples; therefore, it is important to continue efforts to understand better the role of paediatric obesity in sleep health in the Gulf region.

As in adults, obesity in children and adolescents is associated with physical complications, as well as psychosocial issues. The latter manifests in different ways in children and has been investigated to a lesser extent in the Middle Eastern population. While Western samples show an association between paediatric obesity and internalising (e.g., depression) and externalising (e.g., hyperactivity) disorders (17, 40), there was no evidence for an association between weight status and mental health problems in the current sample of UAE youth. However, there was an interaction between risk for experiencing peer problems and sex. For girls, risk for peer problems was elevated for those with higher weight status. This same pattern was observed in the first wave of the US-based Adolescent Brain and Cognitive Development (ABCD) study (40). One explanation may be that girls whose body types diverge from sociocultural norms are more likely to experience weight-related stigma and social exclusion (41). Indeed, a recent qualitative study in the UAE, parents of children with obesity reported concerns related to bullying and social isolation (42). Therefore, it is important to consider the role of social stigma in long-term psychosocial health (43), with particular attention to girls. In the UAE, recent studies show that dietary preferences and eating behaviours are closely linked to body weight, particularly among young women, with higher intake of fast foods and sweetened drinks associated with overweight and obesity (44). A national nutrition review further highlights the ongoing shift toward Westernised diets and lifestyle patterns in the UAE (45). These sociocultural factors may help explain the body-image pressures and peer challenges observed among girls with obesity in this study.

These preliminary findings can have significant implications locally and regionally, as paediatric obesity is increasingly prevalent and relevant in the Middle East. However, the study has a few limitations that must be considered. First, this was a cross-sectional study design with non-random sampling, and therefore may not be representative of all youth in the general population. While recruiting participants exclusively from the ICLDC enabled characterisation of medical records, it might limit the generalizability of the findings to younger individuals who do not seek treatment for a chronic condition. Future studies that incorporate random or school-based sampling across multiple regions of the UAE would yield more representative prevalence estimates.

Additionally, while %IOTF-25 is more closely associated with adiposity than BMI (19), particularly in youth with morbid obesity (18), it is an indirect measure of adiposity and faces similar limitations to other BMI-based metrics (46). While the questionnaires have well-established reliability, sleep and mental health were based on parent report. Parent-reported SDQ scores may under-detect internalising symptoms such as anxiety or depression, particularly among adolescents, who may not express such concerns to parents. Future research should integrate adolescent self-reports to provide a more comprehensive assessment of psychosocial functioning. To draw more definitive conclusions and gain a deeper understanding, larger studies including a broader range of participants are needed. Small subgroup sizes may have limited power to detect modest effects, and the lack of correction for multiple comparisons increases the risk of Type I error. However, the consistency of associations across sensitivity models provides some confidence in the robustness of findings. Therefore, more research is necessary to inform better healthcare plans and interventions that are tailored to the unique needs of different regions and populations.

While there is a need for better powered studies of paediatric obesity in the Gulf region, this preliminary study highlights the importance of considering culturally specific factors. While paediatric obesity is associated with lower socioeconomic status and emotional and behavioural problems in Western samples, these patterns were not observed in Emirati youth.

## 5 Conclusion

This study highlights the need for continued examination of paediatric obesity to support the development of proactive, natively tailored healthcare plans based on a deep understanding of the causal factors in different regions. The recent rise in prevalence in the Gulf region highlights the importance of focusing on specific studies, particularly from the Middle East. Recognition and response to the specific health and social consequences of obesity should be a goal of paediatric obesity interventions and policies in the UAE.

## Data Availability

The datasets presented in this study can be found in online repositories. The names of the repository/repositories and accession number(s) can be found in the article/Supplementary Material.
